# Relations of coping strategies and cognitive emotion regulation to Chinese children’s academic achievement goals and academic performance

**DOI:** 10.3389/fpsyg.2024.1454886

**Published:** 2024-10-22

**Authors:** Li Chen-Bouck, Meagan M. Patterson, Anqi Peng

**Affiliations:** ^1^Graduate School of Education, Baker University, Baldwin City, KS, United States; ^2^Department of Educational Psychology, University of Kansas, Lawrence, KS, United States; ^3^Harmony Institute, Arizona State University, Tempe, AZ, United States

**Keywords:** Chinese children, coping strategies, cognitive emotion regulation, academic achievement goals, academic performance, self-management skills, social and emotional learning

## Abstract

Social–emotional skills (e.g., emotion regulation) influence a variety of academic outcomes among children. This study examined the relations of coping strategies and cognitive emotion regulation (CER) to academic achievement goals and academic performance among Chinese children (*N* = 401, age *M* = 10.52 years). Full Structural Equation Modeling (SEM) analysis showed that children’s use of active coping strategies was positively associated with their endorsement of performance approach and mastery avoidance goals. Avoidant and support-seeking coping strategies were not associated with any outcome variables. Maladaptive CER was positively associated with performance approach, performance avoidance, and mastery avoidance goals and negatively associated with academic performance; adaptive CER was positively associated with mastery approach goals but not associated with other outcome variables. The current study provided some preliminary evidence to indicate that active coping strategies, maladaptive CER, and adaptive CER were associated with academic achievement goals and academic performance among Chinese children. Although maladaptive CER was not used as often as adaptive CER by Chinese children, the more they used maladaptive CER, the more likely they were to have academic achievement goals that focused on things other than learning itself, whereas the more Chinese children used adaptive CER, the more likely they were to set an achievement goal to master the knowledge. Therefore, in teaching practices or intervention planning, decreasing maladaptive CER strategies may be as important as cultivating adaptive CER strategies to facilitate academic success among Chinese children.

## Introduction

Since its introduction in the research literature in 1994, social and emotional learning (SEL) has gained popularity in research and educational practice (Hoffman, 2009). The SEL framework posits the importance of children’s ability to understand and manage their own emotions and interpersonal interactions for their academic success and overall well-being (Jones and Doolittle, 2017), and a growing body of literature links SEL programs to student academic achievement (e.g., Wigelsworth et al., 2016; Zins et al., 2004). The SEL framework views learning as a social process; students learn through collaboration with teachers and peers and emotions can facilitate or hamper learning (Zins et al., 2004). For example, both positive and negative emotions can occupy space in working memory (Pekrun and Stephens, 2009) and potentially reduce available cognitive resources and obstruct learning (Ahmed et al., 2013). In addition, emotions can have both positive and negative impacts on students’ motivational processes (Kwon et al., 2017).

In the SEL framework, one major skill domain is self-management skills, which refers to the ability to successfully regulate one’s own emotions, thoughts, and behaviors and to set and work toward goals (Jones and Doolittle, 2017). Research on relations between self-management skills and academic outcomes has been prevalent for some time, however, most studies focus on participants from Western regions and few studies have examined this topic in China (Chung et al., 2020). Much research has illustrated how Asian and Western cultures have different norms for self-expression and views about self and others (Xu et al., 2006). These distinctive norms may foster a preference for some self-management strategies over others (Xu et al., 2006). Cross-cultural research (e.g., Ramzan and Amjad, 2017) indicates differences in emotion regulation (an element of self-management) between individualistic and collectivistic cultures. For example, members of individualist cultures tend to prefer emotional expression, such as talking with others, to regulate emotions whereas members of collectivistic cultures often prefer expressive suppression (Chen and Kennedy, 2005; Ramzan and Amjad, 2017). Therefore, findings based on Western participants may not apply to the Chinese context.

Late childhood to early adolescence (i.e., mid- to late elementary school years) is a stage when significant changes occur in coping processes and emotion regulation abilities (Zimmer-Gembeck and Skinner, 2011). Developmental changes in coping strategies include shifts in problem-solving (from instrumental action to planful problem solving), greater self-reliance (versus reliance on adults), and greater capacity for using cognitive strategies to cope with stressful events (Zimmer-Gembeck and Skinner, 2011). Similarly, the capacity for emotion regulation develops substantially over the course of the elementary school years, with increasing ability to reduce the intensity of a current emotion and use of cognitive strategies with age (Martin and Ochsner, 2016). As children grow older, the strategies they use for emotion regulation shift from primarily external and behaviorally oriented ones to more internal and cognitively oriented ones, and by 8 or 9 years of age, children have learned to regulate their emotions through cognitive emotion regulation (e.g., Saarni, 1999). However, empirical evidence regarding the specific associations between self-management skills and academic outcomes among children in this particular age period is sparse (Kwon et al., 2017). In addition, given the dual emphasis on academic success and emotional well-being in contemporary China (e.g., Chen-Bouck et al., 2017), it is important to examine the relations between Chinese children’s self-management skills (i.e., coping strategies and cognitive emotion regulation) and their academic outcomes.

### Coping strategies and academic outcomes

Coping refers to the ability to respond to stressful events and regulate one’s thoughts, emotions, and behavior under stress (Ayers et al., 1996). In the current study, coping strategies were examined through a modified four-factor model originally articulated by Ayers et al. (1996). This model includes active, distraction, avoidant, and support-seeking coping strategies. Active coping strategies include doing something or making plans to do something to alter the situation. Distraction coping includes attempts to take one’s mind off of stressful situations through engaging in pleasant activities (e.g., exercise). Avoidant coping involves trying to avoid engaging with or thinking about stressful situations. Support-seeking coping involves reaching out to others, either in the service of talking about feelings or seeking solutions to the problem.

Consistent with the SEL framework, applying social and emotional skills has positive relations to students’ academic outcomes (e.g., better academic performance, more academic motivation, and higher learning goals; Zins et al., 2004). Coping strategies, as one aspect of self-management skills, may relate to students’ academic goals and performance in several ways. First, school may be a source of stressors (e.g., exams, grades) with which students must cope (e.g., Shih, 2018), and pressure for academic success is a noted source of stress among Chinese students (Zhou et al., 2023). Completing academic work outside of school (e.g., homework) is also a potential source of stress for Chinese students given the heavy homework loads assigned by Chinese schools as well as parental engagement with children’s learning activities and school performance (Liu et al., 2019). Second, in addition to coping with academic stressors in particular, broader abilities to cope may promote academic success, whereas a lack of successful coping strategies may interfere with academic engagement and performance as students who lack effective coping skills may be distracted from schoolwork by their emotional reactions or intrusive thoughts (Mantzicopoulos, 1997; Skinner et al., 2016). Third, coping strategies may also relate to academic goals through mechanisms such as perceived competence and self-regulation (Liem, 2022) or promoting a sense of school belonging (Thomas et al., 2023).

Students have different reasons for engaging or not engaging in learning activities, and these reasons are often framed as academic achievement goals (Patrick et al., 2011). One commonly used framework for examining academic achievement goals is Elliot and McGregor’s (2001) four-factor framework, which classifies students’ achievement goals using a 2×2 matrix along the domains of mastery / performance and approach / avoidance. Performance approach goals include the desire to demonstrate one’s competence, achieve high scores or grades, and outperform other students. Performance avoidance goals incorporate the desire to avoid appearing incompetent or being viewed negatively by or relative to others. Mastery approach goals focus on a desire to master content and do one’s personal best within a domain. Finally, mastery avoidance goals emphasize a desire to avoid a lack of mastery or a failure to learn. Meta-analytic findings support the four-factor model and indicate that approach motivations are associated with higher academic achievement and avoidance motivations are associated with lower academic achievement (Huang, 2012).

Most research on the relations between coping strategies and academic achievement goals has examined students’ coping strategies specifically related to academics (e.g., coping with academic failure). For example, a study of Taiwanese eighth graders suggested that engagement coping (e.g., problem-focused coping or cognitive restructuring) was positively associated with both approach and avoidance mastery goals, and disengagement coping (e.g., withdrawal or denial) was negatively associated with mastery approach goals and positively associated with performance avoidance goals (Shih, 2018). Broader coping strategies may also relate to academic motivation through mechanisms like perceived competence and self-regulation (Liem, 2022), however research on the relations between students’ broader coping strategies and their academic achievement goals is comparatively limited. In addition, coping strategies may vary in their effectiveness based on match or mismatch with the demands of particular situations or interactions with other individual or situational variables. For example, Vélez et al. (2016) found that support seeking coping, which is generally considered a positive coping strategy, was associated with negative outcomes (e.g., greater depressive symptoms) for early adolescents who were high in rumination.

The relation of coping strategies to academic performance is understudied relative to relations with other variables; for example, in her review of research on coping strategies in children and adolescents, Clarke (2006) found only 6 studies that examined academic performance as an outcome variable versus 72 studies of internalizing and externalizing problems. However, there is some evidence that coping strategies relate to academic performance (Skinner and Saxton, 2019). For example, empirical studies suggested that positive and action-oriented coping strategies were positively related to intrinsic motivation to learn and academic performance in children (Mantzicopoulos, 1997), engagement coping (or active coping) was related to academic competence in children (Valiente et al., 2009), and disengaged coping (i.e., denial and avoidance of academic problem) was related to lower grade point average (GPA) in adolescents (Arsenio and Loria, 2014). In a meta-analytic review, Clarke (2006) found that the use of active coping strategies was tied to academic performance, although the effect size was small.

### Cognitive emotion regulation and academic outcomes

Cognitive emotion regulation (CER) refers to mental strategies used to handle emotionally arousing information (Garnefski and Kraaij, 2007). CER strategies can be divided into two general categories: maladaptive CER (e.g., blaming self or others, rumination) and adaptive CER (e.g., planning, positive reappraisal; Garnefski and Kraaij, 2007). Some maladaptive CER strategies (i.e., rumination, catastrophizing, and self-blame) are associated with emotional problems across development (Domaradzka and Fajkowska, 2018; Garnefski and Kraaij, 2006), whereas adaptive CER strategies (e.g., positive reappraisal) are associated with positive outcomes, such as resilience following negative life events (Garnefski and Kraaij, 2006). However, several studies have found that culture plays an important role in shaping emotion regulation and emotional experiences by creating value systems that facilitate social norms for regulating emotions (Matsumoto et al., 2008; Miyamoto et al., 2014). For example, the use of emotion regulation strategies such as distancing and shifting focus may contribute to differences in emotional experience in China compared to the U.S. and other Western settings (Davis et al., 2012).

CER may influence students’ academic behaviors and performance in multiple ways (Harley et al., 2019; Kwon et al., 2018; Schlesier et al., 2019; Xu, 2018). First, students experience various emotions when engaging with academic tasks, and many of these emotions (e.g., test anxiety) can interfere with academic performance if not appropriately regulated. For example, inefficient emotion regulation might physiologically inhibit children’s use of higher-order cognitive processes (e.g., the ability to attend to and retain novel information; Blair, 2002). Second, students who lack effective CER strategies may be less likely to persist when faced with academic challenges, whereas adaptive CER strategies may promote students’ persistence in overcoming difficulties. For example, a study examining the role of emotion regulation in mathematics homework among Chinese adolescents suggested that emotion management and cognitive reappraisal were positively related to learning-oriented motivations (Xu, 2018). Third, CER may influence academic performance indirectly through influences on classroom engagement (Kwon et al., 2017), or sleep quality (Tomaso et al., 2021). However, like the research on coping strategies, most existing research on CER in children and adolescents has focused on internalizing and externalizing problems (e.g., Domaradzka and Fajkowska, 2018), rather than academic outcomes.

Although little research has specifically examined the associations between CER and academic achievement goals, research on CER and other academic factors provides relevant findings. For example, in a longitudinal study of U.S. elementary school students, Kwon et al. (2018) found that emotion regulation positively predicted academic engagement 14 months later. Academic engagement is positively associated with academic achievement goals, especially approach goals (Datu and Park, 2019).

Studies suggested that the ability to regulate one’s emotions facilitates functioning in academic contexts (Fried, 2011), whereas the inability to regulate the intensity and duration of one’s emotions will hinder academic performance (Martin and Ochsner, 2016). In order to succeed in school, students must be able to manage and regulate their emotions, including emotions related to academic tasks (e.g., shame after getting a poor grade; Harley et al., 2019) and non-academic events that occur in school (e.g., anger about an argument with a friend; Ben-Eliyahu and Kaplan, 2015). Studies that examined emotion regulation broadly suggested relations between emotion regulation and academic performance in children (e.g., emotion regulation was positively associated with achievement test scores; Schlesier et al., 2019).

### The current study

The SEL framework posits the importance of children’s social and emotional skills in their academic success (Jones and Doolittle, 2017). Prior studies provided evidence for possible associations between coping strategies and CER and children’s academic outcomes, however empirical evidence regarding the specific associations among children in the mid- to late elementary school years is sparse (Kwon et al., 2017), let alone among samples from mainland China. Being informed by the SEL framework, the current study focused, cross-sectionally, on self-management skills, specifically the relations of coping and CER strategies with Chinese children’s academic achievement goals and academic performance.

Regarding coping strategies, we hypothesized that active and support seeking coping strategies would be positively associated with performance approach, mastery approach, and mastery avoidance goals, and academic performance, and negatively associated with performance avoidance goals; avoidant coping strategies would be negatively associated with performance approach, mastery approach, and mastery avoidance goals, and academic performance, and positively associated with performance avoidance goals. Regarding CER, we hypothesized that maladaptive CER would be negatively associated with academic performance, but adaptive CER would be positively associated with academic performance. Given the lack of previous studies, we examined associations between CER and academic achievement goals in an exploratory manner.

## Materials and methods

### Participants

In total 401 children (ages 9–13, *M* = 10.52, *SD* = 0.81; grades 4–6) participated in the study. Among the children, 46.1% were female and 52.6% were male, 1.2% did not identify their gender; 93.8% identified as Han ethnicity and 4% as ethnic minorities, and 2.2% did not identify their ethnicity. Regarding participants’ father’s education level, 1.5% had an elementary level, 26.7% had a secondary level, 59.9% had a college level, 11.0% had a graduate level of education, and 1.0% did not indicate; among mothers, 1.2% had an elementary level, 33.7% had a secondary level, 59.1% had college level, 5.0% had a graduate level of education, and 1.0% did not indicate.

### Procedure

Participants were recruited as part of a larger study of parenting and social, emotional, and academic outcomes for Chinese children [see (Chen-Bouck et al., 2021; Chen-Bouck et al., 2019) for other research drawing on this data set]. Participants were recruited from one elementary school in a large city in northwestern China. Written parental consent and student assent were obtained prior to study participation. The study project was reviewed and approved by the Human Subjects Committee of the University of Kansas.

### Measures

All measures were translated into Chinese using back-translation method (Werner and Campbell, 1970) and bilingual checking (Punnett and Shenkar, 1996) by the first author and two other bilingual researchers, who are fluent in both Chinese and English. Pilot tests were conducted (using an independent sample of 40 children) to test participants’ understanding of the content and wording of the translated Chinese measures. Minor adjustments of the translation were made according to participants’ feedback from the pilot tests.

#### Coping strategies

Participants’ coping strategies were measured with the Children’s Coping Strategies Checklist-3^rd^ Revision (CCSC-R3; Arizona State University Research and Education Advancing Children’s Health (REACH) Institute, 2006). The scale identifies various coping strategies that children use during difficult situations. CCSC-R3 includes 56 items with three subscales. The Active Coping subscale includes two components, problem-focused coping (e.g., *you thought about which things are best to do to handle the problem*) and positive cognitive restructuring (e.g., *you told yourself that you could handle this problem*), with 24 items; the Avoidant Coping subscale has 12 items (e.g., *you tried to ignore it*); and the Support Seeking Coping subscale has 16 items (e.g., *you asked your parents for help in figuring out what to do*). Four items in the CCSC-R3 regarding religion (e.g., *you prayed more than usual*) were excluded in the current study since this type of religious belief is not common in Chinese culture. Items were rated on a 4-point scale ranging from 1 (*never*) to 4 (*most of the time*). For each subscale, a higher mean indicates more frequent use of the specific coping strategy. Ayers et al. (1996) provided evidence supporting the validity of the first version of this scale. In the current sample, the internal consistency coefficients for the Active Coping, the Avoidant Coping, and the Support Seeking Coping subscales were αs = 0.91, 0.78, and 0.85, respectively.

#### Cognitive emotion regulation

Participants’ emotion regulation was measured with the Cognitive Emotion Regulation Questionnaire (CERQ; Garnefski and Kraaij, 2007). The CERQ includes 36 items with nine subscales, however, due to a technical error, the Positive Refocusing subscale was not included in the questionnaire, so eight subscales (32 items) were used in the current study. The eight subscales were: 1. Self-blame (e.g., *I feel that I am the one to blame for it*); 2. Rumination (e.g., *I dwell upon the feelings the situation has evoked in me*); 3. Catastrophizing (e.g., *I keep thinking about how terrible it is what I have experienced*); 4. Other-blame (e.g., *I feel that others are to blame for it*); 5. Acceptance (e.g., *I think that I cannot change anything about it*); 6. Refocus on planning (e.g., *I think about how I can best cope with the situation*); 7. Positive reappraisal (e.g., *I think that the situation also has its positive sides*); 8. Putting into perspective (e.g., *I think that it all could have been much worse*). Response options ranged from 1 (*never*) to 4 (*most of the time*). In the current study, based on the definitions of the eight cognitive coping strategies from the CERQ manual and suggestions from previous studies (Garnefski et al., 2002), subscales 1 to 4 were combined to form a Maladaptive CER Subscale (16 items); and subscales 5 to 8 were combined to form an Adaptive CER Subscale (16 items). For each subscale, a higher mean indicates more frequent use of specific emotion regulation strategies. The original scale is valid and has a relatively high test–retest reliability (Garnefski et al., 2001). In the current sample, the internal consistency coefficients for the Maladaptive CER and Adaptive CER subscales were αs = 0.82 and 0.82, respectively.

#### Academic achievement goals

Participants’ academic achievement goals were measured with the Achievement Goal Questionnaire (AGQ; Elliot and McGregor, 2001). The scale includes 12 items with four subscales (3 items each): Performance Approach Goals (e.g., *it is important for me to do better than other students*), Performance Avoidance Goals (e.g., *I just want to avoid doing poorly in this class*), Mastery Approach Goals (e.g., *I want to learn as much as possible from this class*), and Mastery Avoidance Goals (e.g., *I worry that I may not learn all that I possibly could in this class*). The items were rated on a 7-point scale ranging from 1 (*not at all true of me*) to 7 (*completely true of me*). For each subscale, a higher mean indicates stronger endorsement of the specific achievement goal. The scale is valid and has good internal consistency (Elliot and McGregor, 2001). In the current sample, the internal consistency coefficients for the Performance Approach Goals, Performance Avoidance Goals, Mastery Approach Goals, Mastery Avoidance Goals subscales were αs = 0.61, 0.58, 0.67, and 0.79, respectively.

#### Academic performance

Participants’ academic performance was calculated by averaging scores (0–100 points scale) for the three major subjects in Chinese elementary school (i.e., Chinese, math, and English) for the academic year. A higher mean indicated better academic performance.

### Analytic strategy

The viability of the latent factors was established through the use of confirmatory factor analysis (CFA) measurement models using R. Measurement models were achieved by examining the loadings of the observed variables onto the latent factors. Low factor loading items (i.e., factor loading <0.30; Tavakol and Wetzel, 2020) were excluded from further analysis. Then, based on the results of the measurement models, preliminary analyses were conducted, including descriptive analysis, Pearson correlations for all the measures, one-way repeated measures ANOVA, and paired-samples *t* test using SPSS. SEM was then implemented using R package Lavaan (Rosseel, 2012) to test the hypothesized model on the overall sample. Missing data were accounted for using full information maximum likelihood (FIML) estimation method. Robust maximum likelihood (MLR) estimator was used since the multivariate normality assumption was not met. To assess the overall goodness of fit, we used the chi-square test statistic. We also take the combination of comparative fit index (CFI; >0.90), Tucker–Lewis index (TLI; >0.90), the root mean square error approximation (RMSEA; <0.06), and the standardized root mean square residual (SRMR; <0.08) values as indicating an acceptable fit.

## Results

### Measurement models

The measurement model for coping strategies included 41 of the original 52 items (i.e., Active Coping subscale 22 items, Avoidant Coping subscale 8 items, and Support Seeking Coping subscale 11 items). CFA revealed acceptable model fit, χ^2^(754) = 1101.20, *p* < 0.001, CFI = 0.91, TLI = 0.90, RMSEA = 0.04, 90% CI [0.04, 0.05], and SRMR = 0.06. The measurement model for cognitive emotion regulation included 19 of the original 32 items (i.e., Maladaptive subscale 10 items and Adaptive subscale 9 items). CFA revealed acceptable model fit, χ^2^(143) = 232.13, *p* < 0.001, CFI = 0.95, TLI = 0.94, RMSEA = 0.04, 90% CI [0.03, 0.05], and SRMR = 0.05. The measurement model for academic achievement goals included all 12 original items. CFA revealed acceptable model fit, χ^2^(47) = 118.48, *p* < 0.001, CFI = 0.93, TLI =0.91, RMSEA = 0.06, 90% CI [0.05, 0.08], and SRMR = 0.05. The internal consistency reliability coefficient for each measurement model was examined using R. See Table 1 for reliability coefficients of all the measurement models. See Supplementary Table S1 for factor loadings of items in the measurement models.

**Table 1:** Reliabilities, descriptive statistics, and correlations.

Variable	*α*	*M*	*SD*	1	2	3	4	5	6	7	8	9
1. Active coping strategies	0.91	2.67	0.55									
2. Avoidant coping strategies	0.78	2.14	0.64	0.22***								
3. Support seeking coping strategies	0.85	2.30	0.64	0.57***	0.31***							
4. Maladaptive CER	0.82	2.05	0.72	0.06	0.33***	0.14**						
5. Adaptive CER	0.82	3.22	0.82	0.68***	0.032	0.31***	0.13*					
6. Performance approach goals	0.61	4.80	1.38	0.24***	0.09	0.12*	0.20***	0.14**				
7. Performance avoidance goals	0.58	5.08	1.45	0.19***	0.22***	0.15**	0.27***	0.14**	0.46***			
8. Mastery approach goals	0.67	5.80	1.09	0.31***	−0.01	0.11*	0.06	0.35***	0.47***	0.35***		
9. Mastery avoidance goals	0.79	4.93	1.56	0.19***	0.16**	0.10	0.34***	0.17**	0.42***	0.46***	0.42***	
10. Academic performance		88.94	7.42	0.22***	−0.07	0.02	−0.18***	0.25***	0.06	−0.10	0.15**	−0.12*

M, mean; SD, standard deviation; CER, cognitive emotion regulation.

*p < 0.05; **p < 0.01; ***p < 0.001.

### Preliminary analyses

Regarding the three types of coping strategies, a one-way repeated measures ANOVA was conducted. The results showed that sphericity was violated, therefore the Greenhouse–Geisser adjustment was applied. The corrected degree of freedom and the corresponding *F* value were reported. The one-way repeated measures ANOVA showed significant results, *F* (1.78, 665.79) = 118.24, *p* < 0.001, *η*^2^ = 0.24. Children used active coping strategies most frequently (*M* = 2.67), followed by support seeking (*M* = 2.30), and used avoidant strategies least frequently (*M* = 2.14). Regarding the two types of CER, a paired-samples *t* test indicated a statistically significant difference, *t*(382) = −22.48, *p* < 0.001, *d* = −1.15. Children used adaptive CER (*M* = 3.22) more than maladaptive CER (*M* = 2.05). Regarding academic achievement goals, a one-way repeated measures ANOVA was conducted. The results showed that sphericity was violated, therefore the Greenhouse–Geisser adjustment was applied. The corrected degree of freedom and the corresponding *F* value were reported. The one-way repeated measures ANOVA showed significant results, *F* (2.90, 1102.83) = 69.32, *p* < 0.001, *η*^2^ = 0.15. Among the four achievement goals, children endorsed mastery approach goals most strongly (*M* = 5.80); followed by performance avoidance goals (*M* = 5.08) and mastery avoidance goals (*M* = 4.93) with no difference between the two avoidance goal types; children endorsed performance approach goals (*M* = 4.80) less strongly than performance avoidance goals (*M* = 5.08), but endorsed performance approach goals (*M* = 4.80) and mastery avoidance goals (*M* = 4.93) at the same level. Descriptive statistics and the Pearson correlations for all measures are reported in Table 1.

### Full structural equation model

In the full structural equation model, direct paths were formulated between coping strategies (i.e., active, avoidant, and support seeking coping strategies), cognitive emotion regulation (i.e., maladaptive and adaptive CER), academic achievement goals (i.e., performance approach, performance avoidance, mastery approach, and mastery avoidance goals), and academic performance. Five control variables were included in the model (i.e., age, gender, both parents’ education levels, and family annual income). Correlations among exogenous variables were estimated, and to account for potential relations between endogenous variables, correlations between the disturbance terms among them were also allowed.

**Figure 1:**
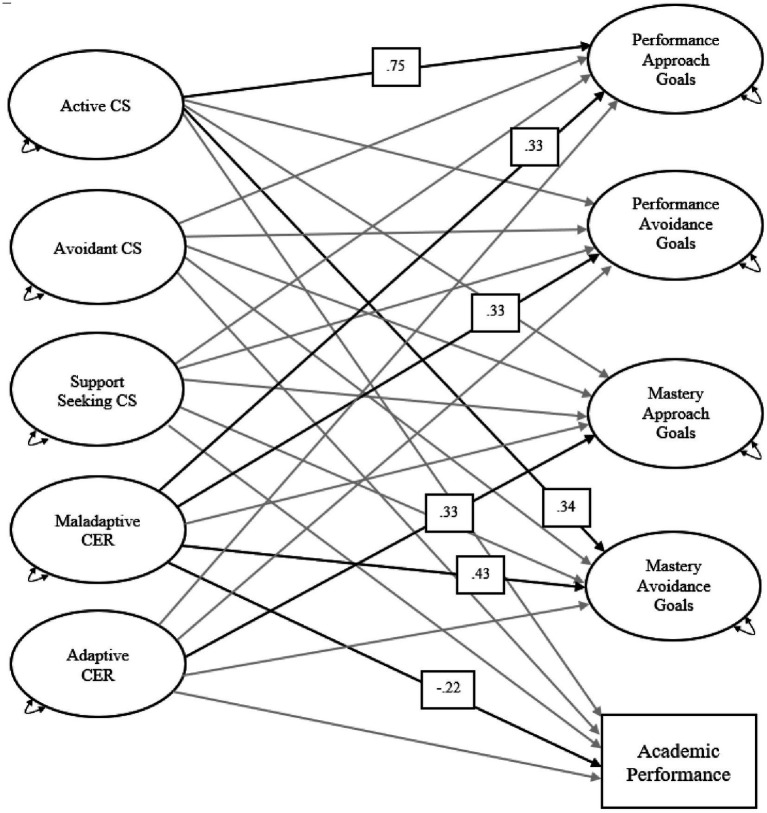
The results of the final structural equation model. Observed variable is denoted by rectangle and latent variables are denoted by ovals. Gray lines represent nonsignificant paths and bold black lines represent significant paths. Standardized coefficients are included for significant paths. Control variables (i.e., age, gender, mother’s education level, father’s education level, and family annual income) were not included in the figure. Correlations among exogenous variables and correlations between the disturbance terms among endogenous variables were not included in the figure. CS, coping strategy; CER, cognitive emotion regulation.

For the sample, the model differed significantly from the data, χ^2^ (2843) = 3857.58, *p* < 0.001, yet fit acceptably according to the Robust CFI = 0.91, Robust TLI = 0.90, Robust RMSEA = 0.03, 90% CI [0.03, 0.03], and Robust SRMR = 0.05. Regarding the associations between coping strategies and outcome variables, the coefficients of the final structural model suggested that active coping strategies were positively associated with performance approach goals (*β* = 0.75, *p* = 0.002) and mastery avoidance goals (*β* = 0.34, *p* = 0.048). Support seeking and avoidant coping strategies were not significantly associated with any outcome variables. Maladaptive CER was positively associated with performance approach goals (*β* = 0.33, *p* = 0.001), performance avoidance goals (*β* = 0.33, *p* < 0.001), and mastery avoidance goals (*β* = 0.43, *p* < 0.001), and negatively associated with academic performance (*β* = −0.22, *p* = 0.002). Adaptive CER was positively associated with mastery approach goals (*β* = 0.33, *p* = 0.027), but was not significantly associated with other outcome variables. See Table 2 and Figure 1 for the results of the full structural equation model.

**Table 2:** Summary of unstandardized, standardized coefficients, and significance levels for the final structural equation model.

Structure model	Unstandardized coefficient (standard error)	Standardized coefficient	*p*
Active coping strategies predicting			
Performance approach goals	0.86 (0.28)	0.75	0.002
Performance avoidance goals	0.34 (0.24)	0.30	0.163
Mastery approach goals	0.30 (0.22)	0.26	0.165
Mastery avoidance goals	0.39 (0.20)	0.34	0.048
Academic performance	0.19 (0.17)	0.18	0.239
Avoidant coping strategies predicting			
Performance approach goals	−0.08 (0.11)	−0.07	0.437
Performance avoidance goals	0.20 (0.11)	0.17	0.062
Mastery approach goals	−0.08 (0.09)	−0.07	0.372
Mastery avoidance goals	0.04 (0.09)	0.03	0.685
Academic performance	−0.01 (0.07)	−0.01	0.919
Support seeking coping strategies predicting			
Performance approach goals	−0.28 (0.17)	−0.24	0.109
Performance avoidance goals	−0.14 (0.17)	−0.12	0.408
Mastery approach goals	−0.14 (0.15)	−0.12	0.350
Mastery avoidance goals	−0.19 (0.14)	−0.16	0.175
Academic performance	−0.13 (0.11)	−0.12	0.239
Maladaptive CER predicting			
Performance approach goals	0.37 (0.11)	0.33	0.001
Performance avoidance goals	0.37 (0.10)	0.33	0.000
Mastery approach goals	−0.04 (0.09)	−0.04	0.646
Mastery avoidance goals	0.49 (0.09)	0.43	0.000
Academic performance	−0.23 (0.08)	−0.22	0.002
Adaptive CER predicting:			
Performance approach goals	−0.38 (0.21)	−0.33	0.065
Performance avoidance goals	0.03 (0.19)	0.03	0.875
Mastery approach goals	0.38 (0.17)	0.33	0.027
Mastery avoidance goals	−0.05 (0.16)	−0.04	0.782
Academic performance	0.19 (0.13)	0.18	0.140

CER, cognitive emotion regulation.

## Discussion

Although the SEL framework posits the importance of children’s social and emotional skills in their academic success (Jones and Doolittle, 2017), empirical evidence regarding the specific impact of these skills among Chinese children in the mid- to late elementary school years is sparse. The current study examined the relations of coping strategies and CER to academic achievement goals and academic performance with a sample of mainland Chinese children (ages 9–13), controlling for children’s age and gender, parents’ education levels, and family income.

### Coping strategies and academic outcomes

Among the three coping strategies, children in our sample used active coping strategies (including problem-focused coping and positive cognitive restructuring) more frequently than avoidant and support-seeking strategies. The findings provide support to the argument that over the course of late childhood and early adolescence, developmental changes in coping strategies include greater self-reliance (versus seeking support from others) and greater capacity for using cognitive strategies to cope with stressful events (Zimmer-Gembeck and Skinner, 2011).

We hypothesized that active coping strategies would be positively associated with performance approach goals, mastery approach goals, mastery avoidance goals, and academic performance, but negatively associated with performance avoidance goals. Results partially confirmed the hypotheses. Active coping strategies were positively associated with performance approach and mastery avoidance goals, but unrelated to the other outcome variables. Our finding is partially consistent with Shih’s (2018) finding that active coping strategies were positively associated with mastery avoidance goals in Taiwanese students. In addition, contrary to our hypotheses, we found no association between either support-seeking coping strategies or avoidant coping strategies and any outcome variable.

In the current study, Chinese children endorsed performance approach and mastery avoidance goals less strongly than the other two goals, however, significant positive correlations were found among all four achievement goals, which was consistent with previous findings that mastery and performance goals are both important to Chinese students and positively correlate with each other in this population (e.g., Shih, 2018). Among the three coping strategies, only active coping strategies were relevant to academic achievement goals. Given these findings, it is unclear why active coping strategies were associated with two less strongly endorsed academic achievement goals, but unrelated to the other two academic achievement goals, further study is needed to confirm this finding.

In addition, no association was found between either support-seeking coping or avoidant coping strategies and outcome variables. In our sample, Chinese children used support-seeking coping and avoidant coping strategies significantly less frequently than active coping strategies. Given the decreased use of support seeking strategies and greater capacity for cognitive coping strategies (e.g., more focus on handling the problem than avoiding the problem) among older children (Zimmer-Gembeck and Skinner, 2011), it is understandable that support-seeking coping and avoidant coping strategies were less clearly tied to achievement goals than were active coping strategies. It is also possible that support seeking coping had positive impacts for some students and negative impacts for others (as in Vélez et al., 2016), leading to an overall lack of observed effects. Thus, more study is needed to verify these findings and further explore the possible mechanisms involved.

Information processing theory posits multiple pathways by which emotion impacts learning and motivation. For example, emotions (e.g., excitement, frustration, hopelessness) can occupy space in working memory during the learning process (Pekrun and Stephens, 2009), and emotions may also distract attention from the learning task, reduce the cognitive resources available, and obstruct learning performance (Ahmed et al., 2013). Children’s emotions can also have positive or negative impacts on their motivational processes (e.g., motivation to learn; Linnenbrink, 2007). Since children’s social–emotional skills and strategies influence their emotions (e.g., if a child can use a coping strategy to effectively handle a negative feeling from a failed test), it could be argued that the associations between coping strategies and academic outcomes could be mediated by variables such as emotions, working memory, or attention. Since this was the first study that examined the specific associations among mainland Chinese children, more studies are needed to confirm the results, and future studies should also consider including mediation analysis to explore the associations further. To shed greater light on the observed associations, more understanding of academic achievement goals among Chinese children is needed. Future research could examine how distinctive the four achievement goal orientations are to Chinese children and how they apply achievement goals in particular academic situations.

### Cognitive emotion regulation and academic outcomes

The current study suggested that Chinese children used adaptive CER (i.e., acceptance, refocus on planning, positive reappraisal, and putting into perspective) significantly more frequently than maladaptive CER (i.e., self-blame, other-blame, rumination, and catastrophizing) with a large effect size. This finding was in line with descriptive statistics presented in previous studies involving both Western and Chinese samples (Garnefski and Kraaij, 2007; Liu et al., 2016); however, none of the studies conducted inferential analyses to examine the difference between the usage of adaptive and maladaptive CER specifically.

Because few studies have examined the associations between CER and academic achievement goals, we examined these relations in an exploratory manner. Maladaptive CER was positively associated with performance approach, performance avoidance, and mastery avoidance goals, and adaptive CER was positively associated with mastery approach goals but not associated with the other three achievement goal orientations. In addition, maladaptive CER was also negatively associated with academic performance. The findings suggested that although maladaptive CER was not used by Chinese children as often as adaptive CER, the more they used maladaptive CER, the more likely they were to care about outperforming others, avoiding appearing incompetent, and avoiding failure to learn, instead of mastering the content—the learning itself. By contrast, the more Chinese children use adaptive CER, the more likely they were to set an achievement goal to master the content. Given that the Chinese education system is highly competitive and academic performance and success are sources of stress among Chinese students (Zhou et al., 2023), it is possible that when Chinese children used maladaptive CER to handle stress from school (e.g., thinking that they were stupid, or thinking it was others’ fault), they were less likely to set an achievement goal that focused on learning itself, whereas when Chinese children used adaptive CER to handle stress (e.g., thinking about learning from it, or thinking about what they can do best), they were more likely to set an achievement goal to master the content and learn better. These findings were consistent with a study with Chinese high school students that cognitive reappraisal, a form of adaptive CER, was positively related to learning-oriented motivations (Xu, 2018). It could be argued that the current findings provided support to the argument that CER contributes to academic outcomes among Chinese children. Taken together, these findings were in line with previous studies that maladaptive CER strategies were associated with negative developmental outcomes whereas adaptive CER strategies were associated with positive outcomes (Domaradzka and Fajkowska, 2018; Garnefski and Kraaij, 2006; Xu, 2018).

### Limitations

There are several limitations in the current study related to measurement. First, after the CFAs, due to low factor loadings, items were excluded from some of the original measures. Thus, it is important to remember that the underlying constructs included in the current study may not exactly parallel those seen in other studies using the same measures. Second, the reliability coefficients were relatively low in three achievement goal subscales, although CFA analysis revealed an acceptable model fit. Descriptive and preliminary analyses revealed that Chinese children endorsed all four achievement goal orientations strongly, with a minimum mean subscale score of 4.80 on a 7-point scale (i.e., *5* means *usually true of me*). Additionally, consistent with the results of a study involving Taiwanese eighth graders (Shih, 2018), all four achievement goal orientations were positively correlated with each other in our sample. Therefore, it is possible that Western constructs of achievement orientations may operate differently in the Chinese context and further examination is required to understand how distinctive the four achievement goal orientations are to Chinese children. Third, the positive refocusing subscale was not included in the measure of CER; thus it is possible that there are effects of adaptive CER driven primarily by positive refocusing (i.e., shifting one’s thoughts away from stressful events and toward more positive thoughts) that would not have been detectable in the current study.

Given the cross-sectional design applied by the current study, future studies with experimental designs (e.g., interventions) and longitudinal data are needed to further verify the findings and examine the impact of coping strategies and CER on academic outcomes. Also, to gain a comprehensive understanding of the mechanisms behind the impact, mediators (e.g., specific emotions, working memory, and attention) should be considered. For example, using experimental or longitudinal designs to examine the impact of a specific coping or CER strategy (e.g., adaptive CER strategies) on children’s academic outcomes. Individual differences should also be considered. For example, previous research indicates that people who are less emotionally aware in general may benefit more from emotion-focused coping interventions (Baker and Berenbaum, 2007). Future research should examine how to tailor coping or cognitive emotion regulation interventions to individual characteristics. In addition, participants were recruited from a large city and the majority of them were from middle class families; further studies are needed to explore the associations among Chinese children from other socioeconomic statuses and settings (e.g., small cities, rural areas). Furthermore, to understand how cultural factors may influence the use and effectiveness of coping strategies and CER, cross-cultural comparison designs should be applied.

### Conclusions and implications

Chinese society has gone through drastic changes in recent decades (Lin et al., 2016). Evidence suggests that while the Chinese education system remains highly competitive and academic success is still a noted source of stress among Chinese students (Zhou et al., 2023), emphasis has shifted from focusing mainly on children’s academic performance to multiple capabilities (e.g., emotional well-being; Chen-Bouck et al., 2017). Under the contemporary cultural and social changes, it is important to understand the possible associations between Chinese children’s social–emotional skills and their academic outcomes, however, research on these specific relations in mainland Chinese settings is limited (Chung et al., 2020). The current study provided some preliminary evidence about the possible associations between Chinese children’s coping strategies and CER and two aspects of their academic outcomes (i.e., academic achievement goals and academic performance). The results suggested that among the three coping strategies, active coping strategies appeared most relevant to Chinese children’s academic achievement goals. Regarding CER, maladaptive CER and adaptive CER were relevant to different academic achievement goals, with adaptive CER associated with goals focused on learning itself and maladaptive CER associated with goals other than learning. Maladaptive CER was also negatively associated with children’s academic performance. However, conclusions about whether the associations of active coping strategies and maladaptive and adaptive CER with any specific academic achievement goal are beneficial or detrimental could be premature. Since achievement orientations may function differently in the Chinese context than in Western settings, without additional empirical evidence on academic achievement goals and how they function among Chinese children, conclusions should not be drawn lightly.

In sum, our findings suggest that to facilitate academic success among Chinese children during late childhood to early adolescence through the use of age-appropriate and effective social and emotional skills, during teaching practice or intervention planning, educators may try to focus on both avoiding or decreasing the use of maladaptive CER strategies and modeling and promoting adaptive CER strategies.

## Data Availability

The raw data supporting the conclusions of this article will be made available by the authors, without undue reservation.
