# Supplementary material for: Relations of coping strategies and cognitive emotion regulation to Chinese children’s academic achievement goals and academic performance
Source: Front Psychol. 2024 Oct 22;15:1454886. doi: 10.3389/fpsyg.2024.1454886 (PMC11534715; doi:10.3389/fpsyg.2024.1454886)
Supplement: Supplementary file 1 [file Table_1.DOCX]

***Supplementary Material***

Table 1

*Summary of Factor Loadings of Items in Measurement Models*

| **Measurement Models** | | Unstandardized coefficient | Standardized coefficient |
| --- | --- | --- | --- |
| **Coping Strategies** ^b^ (41 items) |  | |  |
| Active Coping Strategies (22 items) | |  |  |
| When you had problems, you thought about what you could do before you  did something. | | 1.00ª | .56 |
| You did something to make thing better. | | 0.92 (.13) | .52 |
| You thought about why it happened. | | 0.87 (.14) | .45 |
| When you had problems since last month, you thought about what would  happen before you decided what to do. | | 1.03 (.15) | .52. |
| When you had problems since last month, you tried to make things better  by changing what you did. | | 1.35 (.16) | .69 |
| You tried to understand it better by thinking more about it. | | 1.15 (.16) | .57 |
| You thought about which things are best to do to handle the problem. | | 1.19 (.16) | .59 |
| When you had problems since last month, you did something to solve the  problem. | | 1.30 (.16) | .64 |
| You thought about what you could learn from the problem. | | 1.21 (.15) | .63 |
| You thought about what you needed to know so you could solve the problem. | | 1.26 (.16) | .64 |
| When you had problems since last month, you did something in order to get  the most you could out of the situation. | | 1.25 (.16) | .62 |
| You tried to figure out why things like this happen. | | 1.26 (.16) | .64 |
| You told yourself that you could handle this problem. | | 1.03 (.12) | .56 |
| You told yourself that things would get better. | | 0.91 (.15) | .44 |
| You told yourself you have taken care of things like this before. | | 1.02 (.15) | .50 |
| You told yourself that it would be OK. | | 1.02 (.14) | .53 |
| You told yourself you could handle whatever happens. | | 1.10 (.16) | .53 |
| You told yourself that in the long run, things would work out for the best. | | 1.06 (.16) | .51 |
| You reminded yourself that overall things are pretty good for you. | | 0.99 (.14) | .52 |
| You reminded yourself that you knew what to do. | | 1.31 (.16) | .69 |
| You told yourself that it would work itself out. | | 1.34 (.16) | .66 |
| You reminded yourself about all the things you have going for you. | | 1.18 (.15) | .61 |
| Avoidant Coping Strategies (8 items) | |  |  |
| You tried to ignore it. | | 1.00ª | .63 |
| You tried to stay away from the problem. | | 1.20 (.14) | .75 |
| You imagine how you’d like things to be. | | 0.51 (.11) | .33 |
| You tried to put it out of your mind. | | 1.28 (.14) | .74 |
| When you had problems since last month, you tried to stay away from things  that made you feel upset. | | 0.91 (.12) | .56 |
| You didn’t think about it. | | 0.90 (.12) | .56 |
| When you had problems since last month, you avoided the people who made  you feel bad. | | 0.72 (.12) | .43 |
| You just forgot about it. | | 0.65 (.11) | .44 |
| Support Seeking Coping Strategies (11 items) | |  |  |
| You told your parents how you felt about the problem. | | 1.00ª | .52 |
| You told your parents how you would like to solve the problem. | | 0.76 (.17) | .39 |
| You told your friends about what made you feel the way you did. | | 1.21 (.18) | .61 |
| You talked with friends about what you would like to happen. | | 1.32 (.19) | .66 |
| You told your parents how you felt. | | 0.91 (.13) | .47 |
| You told your brother or sister about what made you feel the way you did. | | 1.00 (.17) | .51 |
| You talked with brother or sister about what you would like to happen. | | 1.01 (.18) | .48 |
| You figured out what you could do by talking with one of your friends. | | 1.19 (.18) | .62 |
| You talked with your brother or sister about your feelings. | | 1.09 (.18) | .53 |
| You talked with your friends about your feelings. | | 1.38 (.20) | .68 |
| You talked to your brother or sister about how to make things better. | | 1.02 (.17) | .51 |
| **Cognitive Emotion Regulation** ^c^ (19 items) | |  |  |
| Maladaptive Cognitive Emotion Regulation (10 items) | |  |  |
| I think that I have been stupid. | | 1.00ª | .54 |
| I think that it’s my own fault. | | 0.92 (.10) | .52 |
| I think that it’s all caused by me. | | 0.92 (.13) | .47 |
| I often think of how I feel about what happened. | | 0.66 (.12) | .33 |
| I often think that it’s much worse than what happens to others. | | 1.21 (.15) | .64 |
| Again and again, I think about how terrible it all is. | | 1.29 (.15) | .72 |
| All the time, I think that this is the worst thing that can happen to you. | | 1.35 (.17) | .67 |
| I often think about how horrible the situation was. | | 1.40 (.17) | .67 |
| I think that it’s the fault of others. | | 0.70 (.11) | .48 |
| I think that others are to blame. | | 0.65 (.10) | .45 |
| Adaptive Cognitive Emotion Regulation (9 items) | |  |  |
| I think about what would be the best for me to do. | | 1.00ª | .49 |
| I think of how I can cope with it. | | 1.38 (.18) | .65 |
| I think of how I can change it. | | 1.34 (.18) | .65 |
| I think of what I can do best. | | 1.25 (.17) | .63 |
| I think that I can learn from it. | | 1.19 (.15) | .56 |
| I think that it makes me feel ‘older and wiser’. | | 1.37 (.18) | .64 |
| I think that there are good sides to it as well. | | 1.16 (.17) | .55 |
| I think that it’s not all bad. | | 0.98 (.16) | .47 |
| I think that I have to accept it. | | 0.91 (.15) | .45 |
| **Academic Achievement Goals** (12 items) | |  |  |
| Performance Approach Goals (3 items) | |  |  |
| It is important for me to do better than other students. | | 1.00ª | .58 |
| It is important for me to do well compared to others in this class. | | 1.04 (.14) | .59 |
| My goal in this class is to get a better grade than most of the other students. | | 0.91 (.12) | .60 |
| Performance Avoidance Goals (3 items) | |  |  |
| I just want to avoid doing poorly in this class. | | 1.00ª | .59 |
| My goal in this class is to avoid performing poorly. | | 0.96 (.14) | .49 |
| My fear of performing poorly in the class is often that motivates me. | | 1.00 (.14) | .57 |
| Mastery Approach Goals (3 items) | |  |  |
| I want to learn as much as possible from this class. | | 1.00ª | .60 |
| It is important for me to understand the content of the class as thoroughly as  possible. | | 1.42 (.17) | .67 |
| I desire to completely master the material presented in the class. | | 1.04 (.13) | .61 |
| Mastery Avoidance Goals (3 items) | |  |  |
| I worry that I may not learn all that I possibly could in this class. | | 1.00ª | .75 |
| Sometimes I’m afraid that I may not understand the content of the class as  thoroughly as I’d like. | | 0.96 (.08) | .71 |
| I am often concerned that I may not learn all that there is to learn in this  class. | | 1.06 (.09) | .78 |

*Note*. ª According to requirements for SEM analyses, one variable loading on each latent factor was set equal

to 1.00 to set the metric for that factor. As a result, significance values are not calculated for these variable

loadings. Standard Errors are in parentheses.

^b^ The stem for Coping Strategies is “*Sometimes you have problems or feel upset about things. When this*

*happens, you may do different things to solve the problems or make yourself feel better*.”

^c^ The stem for Cognitive Emotion Regulation is *“Sometimes nice things happen in your life and sometimes*

*unpleasant things might happen. When something unpleasant happens to you, what do you usually think?”*
